# Fire needle therapy for the treatment of cancer pain: a protocol for the systematic review and meta-analysis

**DOI:** 10.3389/fneur.2024.1358859

**Published:** 2024-09-15

**Authors:** Junning Zhang, Yuehan Ren, Weizhen Wu, Yi Yuan, Jiale Wang, Yi Tang, Yunqiao Zhou, Yizhuo Qiao, Baoqin Liu

**Affiliations:** ^1^Graduate School, Beijing University of Chinese Medicine, Beijing, China; ^2^Department of Oncology of Integrative Chinese and Western Medicine, China-Japan Friendship Hospital, Beijing, China; ^3^School of Acupuncture-Moxibustion and Tuina, Beijing University of Chinese Medicine, Beijing, China; ^4^School of Traditional Chinese Medicine, Beijing University of Chinese Medicine, Beijing, China; ^5^Department of Gynecology, Xiyuan Hospital of China Academy of Chinese Medical Science, Beijing, China; ^6^TCM Gynecology, China-Japan Friendship Hospital, Beijing, China

**Keywords:** fire needle therapy, cancer pain, protocol, systematic review, complementary and alternative medicine, efficacy, safety

## Abstract

**Background:**

Cancer patients frequently suffer pain as one of their symptoms. It includes acute and chronic pain and is one of the most feared symptoms for patients. About one-third of adults actively undergoing cancer treatment suffer from pain related to their condition. Cancer pain control remains suboptimal due to a lack of assessment, knowledge, and access. Fire needle therapy, a traditional Chinese medicine, offers a potentially beneficial addition to current pain management approaches. This protocol outlines a systematic review and meta-analysis to compile evidence and examine the pain-relieving effects and safety of fire needle therapy for cancer patients.

**Methods and analysis:**

We will systematically search China National Knowledge Infrastructure (CNKI), Wanfang Database, China Biology Medicine disc (CBM), China Science and Technology Journal Database (CSTJ or VIP), PubMed, Web of Science, Embase, Cochrane Central Registry of Controlled Trials (CENTRAL), Chinese Clinical Trial Registry (Chictr), Opengrey, Worldcat, and Scopus from inception through July 2023. Random control trials (RCTs) include all types of cancer patients (age ≥ 18 years) complaining of pain. The primary outcome will be changes in pain intensity measured by Visual Analogue Scale (VAS), Numerical Rating Scale (NRS), Neuropathic Pain Scale (NPS), or Brief Pain Inventory (BPI). Secondary outcomes include quality of life (EORTC QLQ-C30 and GCQ), performance status (KPS), times of burst pain, treatment response rate, the dose reduction of analgesic drugs, and side effects rates. Utilizing the Cochrane risk bias measurement tool: Risk of Bias 2 (RoB 2), the trials’ quality will be evaluated, and meta-analysis will be performed using RevMan software (version 5.4).

**Discussion:**

This systematic review will be the first comprehensive review of the literature to provide a meta-analysis of fire needle therapy for cancer pain, including only Random control trials (RCTs). For the sake of transparency and to avoid future duplication, the publication of this protocol offers a clear illustration of the procedures utilized in this evaluation. The results of our future studies may provide a new approach and theoretical basis for the treatment of cancer pain by medical oncology professionals.

**Systematic review registration:**

https://www.crd.york.ac.uk/prospero/, identifier CRD42023418609.

## 1 Introduction

Pain is a prevalent symptom among cancer patients (1). It includes acute pain and chronic pain (2). Cancerous tumors can cause pain by pressing on nerves, bones, or organs, and releasing chemicals that can cause pain (2, 3). The cancer’s destruction of surrounding tissue can also lead to pain (2). Around one-third of adults undergoing active cancer treatment suffer from pain related to their condition (4). Cancer pain can have a significant effect on a patient’s day-to-day activities as well. Cancer pain can lead to sleep disturbances (5), fatigue, nausea, and vomiting (6), all of which can make it difficult to carry out normal activities and enjoy life. Pain can also contribute to feelings of depression and anxiety, further affecting a patient’s quality of life (7). In addition to the physical toll cancer pain takes on patients, it can also have a significant economic burden (8). The cost of pain medications and other treatments can be quite high, and patients may also incur additional expenses related to managing their pain, such as travel to medical appointments or modifications to their home or workplace. This can place a strain on patients and their families, both emotionally and financially (9).

There are many treatments available for pain such as pharmacological agents, nerve blocks, psychological therapies, physiotherapy, alternative remedies, and surgery (10–12). What’s more, a new treatment option, contextual effects (placebo and nocebo effects), should be in the spotlight (13). It has been identified to modulate chronic pain as well as musculoskeletal pain (14, 15). Pain patients use the contextual effect to explain the effects of treatment, while it has been applied in the fields of rehabilitation, physical therapy, and nursing (16–19). For the treatment of cancer pain, the WHO proposed three-step analgesic approach is a major part of this (20). For mild pain, non-opioid analgesics like paracetamol or ibuprofen are used in the first stage; for moderate pain, weak opioids like codeine or tramadol are used in the second step; and for severe pain, strong opioids like morphine or fentanyl are used in the third step (21).

However, there are several restrictions and disadvantages to this strategy. First, due to inadequate opioid prescription or therapy, many patients do not experience sufficient pain relief (22, 23). Second, opioids have serious adverse effects, including addiction, constipation, nausea, drowsiness, and respiratory depression (11, 24). Third, due to supply problems and regulatory restrictions, opioids are frequently unavailable or unaffordable in low- and middle-income nations (25). Consequently, other treatments have been explored and created to decrease reliance on opioids. Fire needle therapy is one of them.

By burning a specific needle till it becomes red and immediately penetrating the skin at the body’s acupuncture point, fire needle therapy is a form of traditional Chinese medicine that heals illnesses (26). Fire needle therapy, which combines acupuncture, direct moxibustion, and needling into one method, is known for its simplicity, practicality, efficiency, and quickness (27, 28). Fire needle therapy has the significant efficacy in treating pain and has sufficient theoretical basis for treating cancer pain (29). According to studies, the use of a fire needle eliminates or improves pathological alterations such as local tissue edema, hyperemia, exudation, and adhesion by stimulating the illness site and reflex point. This boosts metabolism and blood flow while reducing inflammation (30). Fire needle therapy can reduce the levels of pain transmitters in the central and peripheral nerve systems, including substance P and 5-hydroxytryptamine (31, 32). In particular, TNF(tumor necrosis factor) and interleukin-1 levels can both be decreased with fire needle therapy (33). Additionally, it can encourage the body to release more of a vascular endothelial growth factor that is necessary for damage recovery (34). In terms of Traditional Chinese medicine, the acupoints targeted by the fire needle therapy may help to regulate the flow of qi (life force energy) in the body, which could have a positive effect on pain perception (27).

As a result, based on the most recent research, we designed this study to evaluate the efficacy and safety of fire needle therapy in the management of pain in cancer patients. We will also look into the efficient fire needle points and treatment regimens to recommend more usable therapies for clinical care.

## 2 Methods

### 2.1 Study registration

This review protocol is registered in the International Prospective Register of Systematic Reviews (PROSPERO) as CRD42023418609. Reporting standard followed the Preferred Reporting Items for Systematic Reviews and Meta-Analyses (PRISMA) protocols (See Supplementary material).

### 2.2 Eligibility criteria for study inclusion

#### 2.2.1 Type of study

Only RCTs with cancer pain patients treated with fire needle therapy are eligible for inclusion, and there will be no language restrictions during the search process.

#### 2.2.2 Type of intervention

The treatment group will receive fire needle therapy without any restrictions on the depth, frequency, intensity, or area of application of needling. Fire needle therapy either used alone or combined with other therapies (e.g., modern medicine, other acupuncture methods) will be included. RCTs compared fire needle therapy directly with different types of TCM (e.g., herbal decoction, another form of acupuncture) will be excluded from this study.

#### 2.2.3 Type of controls

All the active therapies will be part of the control group. Sham fire needling, no treatment, usual care, oral analgesics, and other active therapies may be included. Placebo, blank controls will be considered for inclusion.

#### 2.2.4 Type of outcome measure

Considering that the population of our study is cancer patients presenting with pain, the results of the evaluation of pain-related scales will be chosen as our primary outcome indicators for analysis, including visual analog scale (VAS), numerical rating scale (NRS), Neuropathic Pain Scale (NPS) and Brief Pain Inventory (BPI). Secondary outcome indicators such as Karnofsky performance status (KPS), the European Organization for Research and Treatment of Cancer Quality of Life Questionnaire (EORTC-QLQC30), the General Comfort Questionnaire (GCQ), times of burst pain, treatment response rate, the dose reduction of analgesic drugs, and side effect rates will be included for analysis. If trials involved any of the above outcome indicators will be included in the analysis.

#### 2.2.5 Type of exclusion criteria

- Duplicate studies;
- Studies that report only abstracts but not complete data;
- Articles with obvious statistical or logical errors.

### 2.3 Search methods for identification of studies

#### 2.3.1 Electronic data sources

The following databases will be searched from their inception through July 2023: China National Knowledge Infrastructure (CNKI), Wanfang Database, China Biology Medicine disc (CBM), China Science and Technology Journal Database (CSTJ or VIP), PubMed, Web of Science, Embase, Cochrane Central Registry of Controlled Trials (CENTRAL), Chinese Clinical Trial Registry (Chictr), Opengrey, Worldcat, and Scopus.

#### 2.3.2 Searching other resources

Any eligible studies potentially overlooked will also be manually identified by scanning the reference lists of related systematic reviews and conference proceedings.

#### 2.3.3 Search strategy

See Supplementary material for details.

### 2.4 Data collection and analysis

#### 2.4.1 Selection of studies

**Figure 1:**
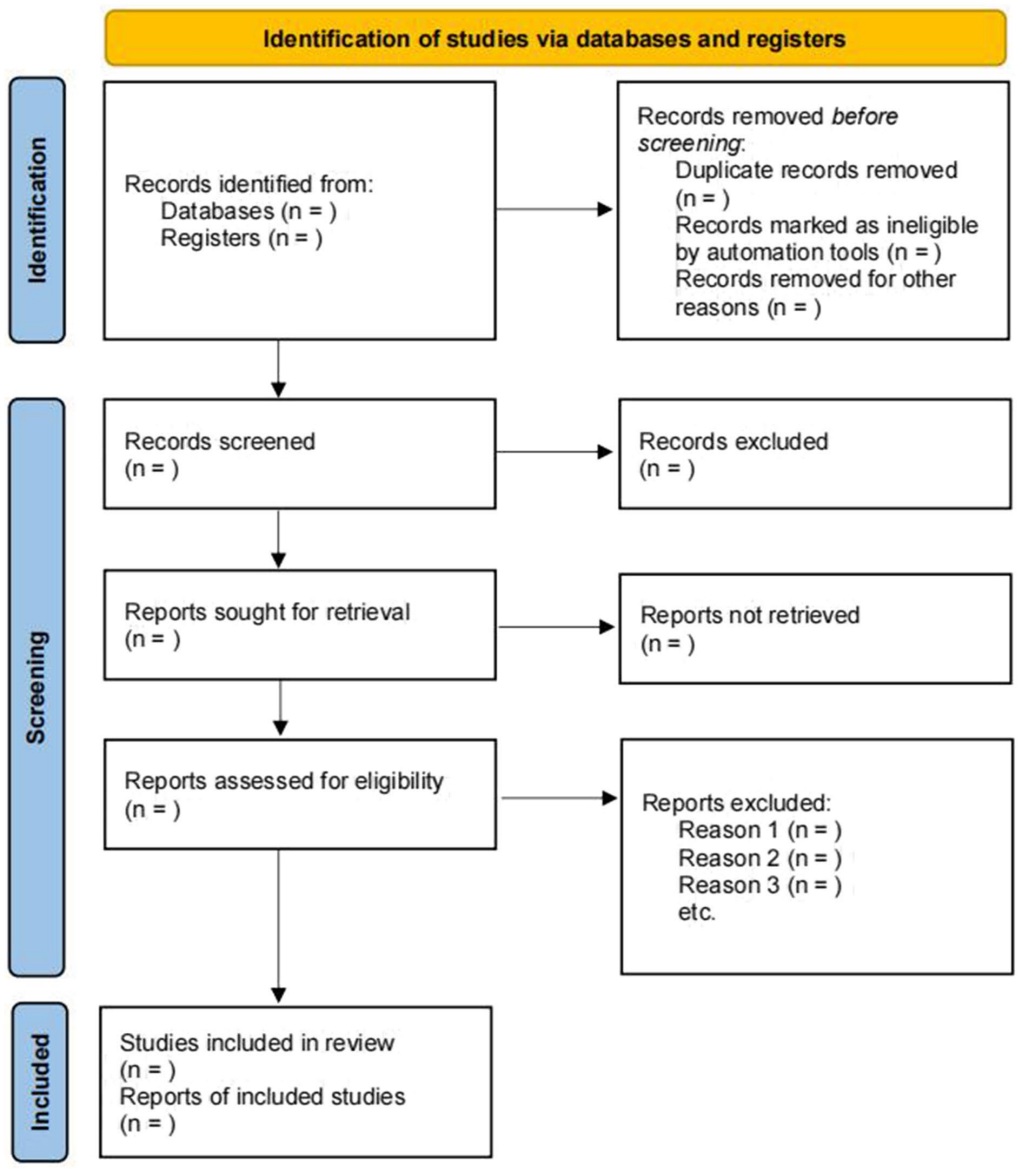
Flowchart of the literature review and selection process (35).

Duplicates were found and eliminated using Zotero software after gathering the search results from these databases. Then, two independent reviewers (JN Z and YH R) will browse the titles and abstracts of the studies, and studies that do not meet the eligibility criteria based on the titles and abstracts will be excluded. The remaining studies’ complete texts will be located and reviewed for the last round of selection. The arbiter (WZ W) shall resolve any differences of opinion on the data selection. The method for finding and screening studies will be depicted in a PRISMA flow diagram (see Figure 1).

#### 2.4.2 Data extraction and management

Two reviewers JN Z and YH R will use a standardized data extraction form to extract the following information for each included RCT:

- Basic information about the RCT: such as title, authors, date of publication, and number of subjects involved;
- Criteria for inclusion of subjects: such as restrictions on demographic information, diagnostic criteria for disease, age, sex, ethnicity, the severity of disease, and duration of disease;
- Details of interventions and controls: such as the method of fire needle therapy, site of acupuncture, dose, duration of treatment, and combined intervention regimen;
- Outcome indicators: names, definitions, and results of outcome indicators;
- Methodology entries: randomized methods, blinded methods (including blinding of physicians, patients, and outcome assessors), allocation concealment, missing data, selective reporting.

A third investigator (WZ W) will make judgments about any differences and questions. Following a transfer of data, the synthesis will be performed using RevMan software (version 5.4, Cochrane Collaboration, Oxford, United Kingdom) (36).

#### 2.4.3 Assessment of risk of bias in the included studies

Two authors (JN Z and YH R) will independently evaluate the risk of bias using the Cochrane risk bias measurement tool: Risk of Bias 2 (RoB 2). The following biases will be assessed. The tool consists of the following 5 domains, randomization process, deviations from intended interventions, missing outcome data, measurement of the outcome, and selection of the reported result. The authors classified studies as low risk of bias, some concerns or high risk of bias.^1^ The arbiter (WZ W) will resolve any disagreements regarding the bias assessment. The quality assessment results will be illustrated in a risk-of-bias graph and a risk-of-bias summary table.

#### 2.4.4 Data synthesis

Data synthesis for the meta-analysis will be accomplished using the RevMan version 5.4 software. Referring to Chapter 10 of the Cochrane Handbook for Systematic Reviews of Interventions,^2^ we chose to use a fixed-effects model or a random-effects model based on the magnitude of heterogeneity. When heterogeneity was significant (I square ≥ 50%, or *p* < 0.05), a random-effects model was used; when it was not significant (I square < 50% and *p* ≥ 0.05), a fixed-effects model was used. A 95% confidence interval (CI) will be used to determine the mean difference (MD) for continuous data. The risk ratio (RR) with 95% confidence interval (CI) will be utilized for dichotomous data. Weighted mean difference (WMD) will be used in subgroup analyses comparing different treatments, and standardized mean difference (SMD) will be used in subgroup analyses comparing the efficacy of the same treatment in different populations.

#### 2.4.5 Dealing with missing data

If the full text of an article is not available, the reviewers will attempt to contact the author to obtain it. If the author cannot be contacted, this article will be excluded from the analysis. The state of relevant articles for which the authors were contacted will be reported in the study results and provide a descriptive summary of the main results of the relevant articles.

#### 2.4.6 Assessment of heterogeneity

For heterogeneity among the outcomes of the included studies, the Chi2 test will be used. The following *I^2^* thresholds will be applied in this study:

- *I^2^*:0–40%: probably not significant;
- *I^2^*:30–60%: possibly moderate heterogeneity;
- *I^2^*:50–90%: possibly significant heterogeneity;
- *I^2^*:75–100%: possibly substantial heterogeneity.

The statistical value of *I^2^* will depend on the magnitude of its influence factors and the strength of evidence of heterogeneity (such as the *p*-value of the Chi^2^ test). The effects of clinical and statistical heterogeneity will be addressed when examining the analysis results (37).

A random-effects model will be applied if significant statistical heterogeneity is discovered; otherwise, a fixed-effects model will be applied. Also, we will assess clinical heterogeneity, including study population, study design, and treatment protocols. The results of the synthesis will be visualized in the form of a forest plot. If heterogeneity is too great, quantitative analysis will be abandoned in favor of qualitative analysis.

#### 2.4.7 Assessment of publication biases

If over 10 RCTs are included in the meta-analysis, publication bias will be evaluated using funnel plots. If there are less than 10 RCTs in the meta-analysis, we will employ Egger’s test and Begg’s test for publication bias.

#### 2.4.8 Subgroup analysis

If the data permits, we will perform the following subgroup analyses:

- The depth, frequency, and intensity of different piercings;
- Cancer pain of the patient (different cancers).

#### 2.4.9 Sensitivity analysis

To assess the impact of study design, sample size, and methodological quality, a sensitivity analysis will be performed and, where possible, the robustness of the data synthesis will be determined.

### 2.5 Grading the quality of evidence

Considering the type of study we included as a randomized controlled trial, randomized controlled trials should be initially rated as high-quality evidence according to the GRADE approach. However, due to limitations in five aspects, the quality of evidence may be downgraded to some extent. These five aspects include: study design limitations (downgrade 1–2 levels), inconsistency of results (downgrade 1–2 levels), indirectness (downgrade 1 level), imprecision (downgrade 1 level), and publication bias (downgrade 1 level). The degree of the downgrade will be determined with reference to the Chapter V of the *Cochrane Handbook for Systematic Reviews of Interventions* (38), and related literature (39).

### 2.6 Patient and public involvement

This meta-analysis data processing phase will utilize published clinical trial data without direct patient or public participation.

### 2.7 Dissemination and ethics

Because the data used in the study are not individualized, ethical approval is not required for this study. Necessary protocol revisions will be documented in a comprehensive review. The study findings will be published in peer-reviewed journals and potentially presented at applicable conferences.

## 3 Discussion

In recent years, due to changes in living environment, the incidence of cancer continues to rise and about 69% of patients suffer cancer pain, including pain caused by cancer, cancer-related, lesions and anti-cancer treatment (40). Cancer pain can occur at any stage from early to late stage of cancer, seriously affecting patients’ treatment and daily life (41). The WHO’s three-step analgesic ladder for cancer pain control is the primary approach for managing cancer pain, with opioids as the core component of the analgesia. However, long-term use of opioids can cause adverse reactions such as constipation, nausea, and vomiting, as well as many problems such as addiction, dependence, and poor tolerance (42). Compared with Western medicine, acupuncture therapy in the treatment of cancer pain is not only effective but also has the advantages of safety, simple operation, small adverse reactions, no dependence, and addiction (43).

Research has shown that pain relief from acupuncture is associated with neuro-humoral factors (44), which can relieve pain by encouraging the release of endogenous opioid peptides, increasing local endorphin levels and peripheral opioid receptor activity during inflammatory responses, and suppressing the synthesis of endogenous pain (45, 46). The stimulation amount of fire needle is much greater than that of traditional acupuncture (29). Thus, fire needle therapy may be able to treat pain that cannot be relieved by ordinary acupuncture (47). Several studies have found that fire needle alone or combined with Western medicine can significantly relieve the pain symptoms of cancer pain patients and reduce the NRS score (47, 48). Compared to using only Western medicine, fire needling alone or in combination with Western medicine demonstrates higher efficacy and lower incidence of adverse effects (48–51). In conclusion, fire needle is an appropriate treatment for cancer pain and should be gradually promoted in clinical practice.

There are some limitations of fire needle therapy. Firstly, there is a fearfulness in patients and a poor reception of that. Secondly, the efficacy and safety of fire-needle therapy need further research and proof. Thirdly, there are potential risks including pain, skin lesions, allergy, and other adverse reactions (52, 53).

This review aims to present information on the different acupuncture points and fire needle therapy that can be used to treat pain in cancer patients. It will cover more than just the effects of fire needling on symptoms in patients with cancer pain. This study may have practical implications for utilizing fire needle therapy in oncology. It could help guide the application of fire needling as an alternative treatment for cancer pain management, thus helping clinicians determine whether fire needle therapy is an effective option to incorporate into pain management programs for cancer patients.

### 3.1 Limitations

However, this study has many limitations. Additional factors that could influence the results include the participants’ experience and expectations with acupuncture, the potential diversity of individualized treatments, and the minimization of inclusion and exclusion criteria (54). In addition, the current study may have some methodological or research quality problems, which may lead to the fact that the exactness of the evidence that we end up with in our study may not be very high. To solve the above problems, we will set strict inclusion and exclusion criteria in the literature screening and data extraction stage, and try to select only the higher quality-studies. Quality assessment will be conducted to differentiate the quality of studies. We will also improve the certainty of evidence by designing and implementing high-quality original studies when conditions permit. In the meantime, we will call for more high-quality studies of RCTs, pending further evidence accumulation.

## Author contributions

JZ: Conceptualization, Formal analysis, Investigation, Writing – original draft, Writing – review & editing. YR: Software, Writing – original draft, Writing – review & editing. WW: Data curation, Writing – original draft, Writing – review & editing. YY: Writing – original draft, Writing – review & editing. JW: Writing – review & editing. YT: Writing – review & editing. YZ: Writing – review & editing. YQ: Writing – review & editing. BL: Funding acquisition, Writing – original draft, Writing – review & editing.

## Footnotes

^1^ https://training.cochrane.org/handbook/current/chapter-08

^2^ training.cochrane.org/handbook/current/chapter-10
