# Supplementary material for: Fire needle therapy for the treatment of cancer pain: a protocol for the systematic review and meta-analysis
Source: Front Neurol. 2024 Sep 15;15:1358859. doi: 10.3389/fneur.2024.1358859 (PMC11438586; doi:10.3389/fneur.2024.1358859)
Supplement: Supplementary file 2 [file Table_1.docx]

Supplementary Material

**Fire needle therapy for the treatment of cancer pain: a protocol for the systematic review and meta-analysis**

**Search strategy**

| Database | No | Search Terms |
| --- | --- | --- |
|  |  |  |
| China National Knowledge Infrastructure (CNKI) | 1 | TKA%=('火针' + '火%针') AND TKA%=('癌痛' + '癌#痛' + '瘤#痛') AND TKA%=('临床#试验' + '临床#疗效' + '随机' + '对照' + '安慰' + '分组' + '随机对照#试验' + '随机对照#研究') AND TKA%=('人') NOT TKA%=('动物' + '鼠' + '兔') |
| Wanfang Database | 1 | 主题:(火针) and 主题:(癌痛) and 主题:(临床疗效 or 临床试验 or 随机对照研究 or 安慰 or 分组) and (摘要:(人） or 题名或关键词:(人)) not (主题:(动物 or 鼠 or 兔) or 题名或关键词:(动物 or 鼠 or 兔)) |
| VIP | 1  2  3  4  5  6 | \| 篇关摘：火针 \| \| --- \| \| 篇关摘：癌痛 + 瘤痛 \| \| 篇关摘：临床疗效 or 临床试验 or 随机对照研究 or 安慰 or 分组 \| \| 篇关摘：人 \| \| 篇关摘：动物 or 鼠 or 兔 \| \| #1 and #2 and #3 and #4 not #5 \| |
| CBM | 1 | "火？针"[常用字段:智能] OR "火针"[常用字段:智能] AND( "癌痛"[常用字段:智能] OR ( "癌？痛"[常用字段:智能]AND "瘤？痛"[常用字段:智能])) 限定条件： 临床试验; 随机对照试验; |
| Web of Science | 1 | (TS=(cancer pain) OR TS=(neoplasm pain) OR TS=(tumor pain) OR TS=(tumour pain)) AND (TS=(fire needling) OR TS=(fire needle) OR TS=(burning needle) OR TS=(hot needle)) AND (TS=(randomized controlled trial) OR TS=(controlled clinical trial) OR TS=(randomized) OR TS=(randomly) OR TS=(trial) OR TS=(placebo) OR TS=(groups) OR KP=(trial)) AND TS=(humans) NOT (TS=(animals) OR TS=(mouse) OR TS=(rabbit)) |
| Embase | 1  2  3  4  5  6 | \| 'cancer pain':ti,ab,kw OR 'neoplasm pain':ti,ab,kw OR'tumour pain':ti,ab,kw OR 'tumor pain':ti,ab,kw \| \| --- \| \| fire needling':ti,ab,kw OR 'fire needle':ti,ab,kw OR burning needle':ti,ab,kw OR'hot needle':ti,ab,kw \| \| 'randomised controlled trial':ti,ab,kw OR 'controlled clinical trial':ti,ab,kw OR 'randomised':ti,ab,kw OR 'randomly':ti,ab,kw OR 'trial':ti,ab,kw OR 'groups':ti,ab,kw OR 'placebo':ti,ab,kw \| \| 'humans'/exp \| \| 'animals'/exp \| \| #1 AND #2 AND #3 AND #4 NOT #5 \| |
| Cochrane Central Registry of Controlled Trials (CENTRAL) | 1  2  3  4 | \| 'cancer pain':ti,ab,kw OR 'neoplasm pain':ti,ab,kw OR'tumour pain':ti,ab,kw OR 'tumor pain':ti,ab,kw \| \| --- \| \| fire needling':ti,ab,kw OR 'fire needle':ti,ab,kw OR burning needle':ti,ab,kw OR'hot needle':ti,ab,kw \| \| 'controlled':ti,ab,kw OR 'randomised':ti,ab,kw OR 'randomly':ti,ab,kw OR 'groups':ti,ab,kw OR 'placebo':ti,ab,kw \| \| #1 AND #2 AND #3 \| |
| PubMed | 1  2  3  4  5 | \| cancer pain OR neoplasm pain OR tumour pain OR tumor pain \| \| --- \| \| fire needling OR fire needle OR burning needle OR hot needle \| \| randomised controlled trial[pt] OR controlled clinical trial[pt] OR randomised[tiab] OR randomly[tiab] OR trial[tiab] OR groups[tiab] OR placebo[tiab] \| \| humans[mh] NOT animals[mh] \| \| #1 AND #2 AND #3 AND #4 \| |
| Chictr | 1 | 注册题目：火针 or 火鍉针 or 火刃针 研究类型：干预性研究/Interventional or 治疗研究/Treatment study |
| ClinicalTrials | 1 | Condition/disease: cancer pain OR neoplasm pain OR tumour pain OR tumor pain Intervention/treatment: fire needling OR fire needle OR hot needle OR burning needle |
| Opengrey | 1 | (cancer pain OR neoplasm pain OR tumor pain OR tumour pain) AND (fire needling OR fire needle OR burning needle OR hot needle) AND (randomized controlled trial OR controlled clinical trial OR randomized OR randomly OR trial OR placebo OR groups) NOT (animals OR mouse OR rabbit) |
| Worldcat | 1  2  3  4  5 | \| Subject=cancer pain OR neoplasm pain OR tumour pain OR tumor pain \| \| --- \| \| Subject=fire needling OR fire needle OR burning needle OR hot needle \| \| Subject=randomized controlled trial OR controlled clinical trial OR controlled OR randomised OR randomly OR groups OR placebo \| \| Subject=animals OR mouse OR mice OR rabbit \| \| #1 and #2 and #3 not #4 \| |
| Scopus | 1 | (TITLE-ABS-KEY=(cancer pain) OR TITLE-ABS-KEY=(neoplasm pain) OR TITLE-ABS-KEY=(tumor pain) OR TITLE-ABS-KEY=(tumour pain)) AND (TITLE-ABS-KEY=(fire needling) OR TITLE-ABS-KEY=(fire needle) OR TITLE-ABS-KEY=(burning needle) OR TITLE-ABS-KEY=(hot needle)) AND (TITLE-ABS-KEY=(randomized controlled trial) OR TITLE-ABS-KEY=(controlled clinical trial) OR TITLE-ABS-KEY=(randomized) OR TITLE-ABS-KEY=(randomly) OR TITLE-ABS-KEY=(trial) OR TITLE-ABS-KEY=(placebo) OR TITLE-ABS-KEY=(groups)) AND TITLE-ABS-KEY=(humans) NOT (TITLE-ABS-KEY=(animals) OR TITLE-ABS-KEY=(mouse) OR TITLE-ABS-KEY=(rabbit)) |

China National Knowledge Infrastructure (CNKI): TKA=title OR keyword OR abstract, %= 'str' indicates that the relevant record matches str.

Web of Science: TS=title OR abstract OR keyword.

Embase: ti=title, ab=abstract, kw=keyword, exp=Emtree term (similar to Mesh term in Pubmed).

Cochrane Central Registry of Controlled Trials (CENTRAL): ti=title, ab=abstract, kw=keyword.

Scopus: TITLE-ABS-KEY=title OR abstract OR keyword.
